# Identification of Important Nodes Based on Local Effective Distance-Integrated Gravity Model

**DOI:** 10.3390/e27040408

**Published:** 2025-04-10

**Authors:** Sheng Zhang, Fuhao Liu, Yuyuan Huang, Ziqiang Luo, Ka Sun, Hongmei Mao

**Affiliations:** School of Information Engineering, Nanchang Hangkong University, Nanchang 330063, China; 2304085410011@stu.nchu.edu.cn (F.L.); 2304081200001@stu.nchu.edu.cn (Y.H.); 2304085410004@stu.nchu.edu.cn (Z.L.); 70308@nchu.edu.cn (K.S.); maohongmei@nchu.edu.cn (H.M.)

**Keywords:** complex networks, node importance, effective distance, fusion gravity

## Abstract

The research into complex networks has consistently attracted significant attention, with the identification of important nodes within these networks being one of the central challenges in this field of study. Existing methods for identifying key nodes based on effective distance commonly suffer from high time complexity and often overlook the impact of nodes’ multi-attribute characteristics on the identification outcomes. To identify important nodes in complex networks more efficiently and accurately, we propose a novel method that leverages an improved effective distance fusion model to identify important nodes. This method effectively reduces redundant calculations of effective distances by employing an effective-influence node set. Furthermore, it incorporates the multi-attribute characteristics of the nodes, characterizing their propagation capabilities by considering local, global, positional, and clustering information and thereby providing a more comprehensive assessment of node importance within complex networks.

## 1. Introduction

Research on complex networks has always been subject to widespread attention. Complex networks can effectively describe and represent large-scale complex systems found in the world, such as biological systems [1,2], medical systems [3], power systems [4,5], and social systems [6,7]. In addition, identifying important nodes in complex networks has applications for various fields. In the field of biology, the identification of important nodes can help reveal key genes, proteins, or other biological molecules, thereby deepening our understanding of the key functions and regulatory mechanisms in biological systems [8]. The identification of important nodes helps to identify and control key spreaders involved in the spread of infectious diseases, thereby effectively formulating intervention strategies and preventive measures [9]. In the maintenance of power systems, the identification of important nodes helps to optimize the stability, reliability, and efficiency of power networks, as well as effectively manage energy distribution and supply strategies [10]. In curbing the spread of rumors, the identification of important nodes helps to identify and control key spreaders in rumor dissemination, allowing one to effectively prevent and respond to the spread of rumors [11].

There are many existing methods for identifying important nodes in complex networks. Traditional methods for identifying important nodes are based on the local and global information of the network, such as degree centrality [12] and K-Shell centrality [13]. The degree centrality method posits that the more neighbors a node has, the more important that node is. The K-Shell centrality method, on the other hand, suggests that a node’s position and hierarchical structure within the entire network significantly influence its importance, with nodes closer to the network core being considered more important. Although traditional methods have achieved good results in some respects, they still have many shortcomings.

Some researchers have proposed the random walk method [14], inspired by natural phenomena such as Brownian motion and molecular dynamics. Its operation logic is as follows: Firstly, a starting node is selected, from which a neighbor node is randomly selected and moved to according to a specific probability distribution, and access information is recorded at the same time. This process is repeated until a preset number of steps is reached or the termination conditions are met. Finally, the importance of the nodes is evaluated through the statistical analysis of their access frequency, coverage time, and other indicators. This method provides a dynamic and global perspective for the identification of key nodes in complex networks, effectively making up for the shortcomings of traditional static methods, and shows unique advantages in certain structures such as planar networks and uniform node-degree networks.

In recent years, gravity model-based methods [15] for identifying important nodes in complex networks have been proposed. This approach leverages the universal law of gravitation, treating a node’s degree value as its ‘mass’ and the shortest path between nodes as the ’distance’ between them, and calculates the force between nodes as an estimate of node importance. Compared to traditional methods, the gravity model-based approach can more accurately capture the complex relationships and interactive influences between nodes, resulting in more precise outcomes. Gravity model methods [16] based on effective distance provide an innovative solution for identifying important nodes in complex networks by treating the effective distance as the distance between nodes and the degree of the nodes as their quality. It is believed that the effective distance can uncover the hidden dynamic structure and dynamic interaction information between nodes, which includes the way the network actually operates, while combining dynamic and static information to identify important nodes can improve the accuracy of the results. The generalized gravity model [17] provides an effective tool for identifying important nodes in complex networks by taking the shortest distance between nodes as the distance and their propagation capacity as their mass. The propagation capability of a node is represented by that node’s local clustering coefficient and degree. Li Hanwen et al. [17] argue that if nodes have the same degree, the node with a higher local clustering coefficient, that is, the node with more edges connected to neighboring nodes, has a stronger ability to propagate information; thus, the propagation capability of a node can more accurately measure the local information of a node.

In summary, previous research on methods for identifying key nodes has analyzed node interactions from various perspectives, thereby providing a more comprehensive assessment of node importance. However, these methods have not yet fully leveraged the multi-scale characteristics of nodes for in-depth analyses. Consequently, this study proposes a novel approach, which we term the local effective distance-integrated gravity model (LEDGM). LEDGM is rooted in the recognition that nodes in complex networks possess intricate relationships that extend beyond their immediate connections. Our approach is anchored in the belief that a holistic analysis, which considers the multifaceted nature of nodes, is essential for accurately capturing their true influence within a network. By integrating various attributes such as local, global, positional, and clustering information, our model endeavors to paint a more nuanced picture of each node’s role and potential impact. This comprehensive assessment allows for a more precise identification of the key nodes that are pivotal to a network’s structure and function. The LEDGM is designed to bridge the gap between traditional methods and the complex reality of network dynamics, providing a framework that is both sophisticated and adaptable to the nuances of different network topologies. Our main contributions are as follows:

(1) We propose a novel approach called the local effective distance-integrated gravity model. This model is specifically designed to offer a more comprehensive assessment of a node’s spreading capability and significance. It incorporates several crucial pieces of information about the nodes, including their local and global characteristics, their positions within the network, and their clustering behavior. By taking all these factors into account, our model provides a more nuanced understanding of each node’s role and influence within the network. This enables researchers and practitioners to identify important nodes with greater precision, which is essential for various applications such as targeted interventions, information dissemination strategies, and network resilience enhancement.
(2) We propose a method that is based on an effective-influence node set. It can adaptively determine the number of nodes needed to be considered according to the network topology, thus effectively improving the algorithm’s efficiency and accuracy.

The rest of this paper is organized as follows: We present the relevant research in Section 2, including a series of foundational studies and centrality measurement methods. The improved effective distance-integrated gravity model proposed in this paper is introduced in detail in Section 3. In Section 4, we will demonstrate the effectiveness of this method through multiple experiments and analyze the experimental results, before summarizing this paper in Section 5.

## 2. Preliminaries

Given an undirected graph G = (V,E), where V represents the set of nodes and E represents the set of edges, the number of nodes in the graph is denoted by N, where N = |V|. The adjacency matrix of graph G is denoted as A = ${{(a}_{ij})}_{N*N}$, where $a_{ij}$ = 1 indicates that there is an edge between node i and node j and $a_{ij}$ = 0 indicates that there is no edge between node i and node j. Additionally, $d_{ij}$ represents the shortest distance between node i and node j, that is, the length of the path from one node to another with the least number of sides.

### 2.1. Related Research

#### 2.1.1. Effective Distance (D)

Effective distance [18] is a concept abstracted from probability that represents the true distance between two nodes instead of the shortest distance. If node i is directly connected to node j, the effective distance from i to j is given by

(1) $$D_{j|i}=1-\log_{2}P_{j|i}$$

(2) $$P_{j|i}=\frac{a_{ij}}{k_{i}}(i\neq j)$$

where $P_{j|i}$ is the probability of node i reaching node j, $a_{ij}$ is the element in the adjacency matrix of graph G, and $k_{i}$ denotes the degree of node i. For nodes that are not directly connected, their effective distance can be obtained through transitivity. If there are multiple paths from node i to node j, the shortest path between the two nodes is taken as their effective distance.

#### 2.1.2. Local Clustering Coefficient (C)

The local clustering coefficient [19] is a measure of the degree to which nodes connected to a particular node are also connected to each other. It describes the density of connections between the neighbors of a node, that is, the extent to which nodes in the local sub-graph centered on a node form closed triangles. A high local clustering coefficient indicates that the neighbors of a node are more likely to be connected to each other. The specific formula is as follows:

(3) $$C_{i}=\frac{2n_{i}}{k_{i}\times (k_{i}-1)}$$

where $k_{i}$ represents the degree of node i and $n_{i}$ represents the number of edges between the neighbors of node i.

#### 2.1.3. Truncation Radius (R)

The truncation radius [20] is a concept used within complex networks that usually refers to the average shortest path length from one node to other nodes in the network, considering only paths with lengths that are not greater than a certain truncation value R. It is used to describe the local connectivity characteristics between nodes in a network, playing an especially important role in large-scale networks due to the extensive computational requirements involved in determining the network’s truncation radius. It was discovered through extensive experiments that the algorithm performs optimally when R is set to half the diameter of the network.

#### 2.1.4. Effective-Influence Node Set (φ)

In previous studies, when employing a gravitational model based on effective distance to calculate the centrality index of nodes, researchers typically considered all nodes in the network. However, this approach is not appropriate because the influence of a node on distant nodes is usually negligible, and such redundant calculations can lead to distorted results and reduced computational efficiency. Research by Li Zhe et al. [20] has shown that using a truncated radius in a gravitational model to assess the importance of nodes can significantly reduce the time complexity of the calculations required and enhance the precision of such experiments. Subsequently proposed gravitational models have largely adopted the concept of the truncated radius R.

Nevertheless, the calculation of the effective distance is costly, and directly comparing the effective distance between nodes with the truncated radius is not practical. To address this issue, we introduce the concept of an effective-influence node set. According to previous studies, the shortest distance can serve as a measure of the distance between nodes, while the effective distance can reveal hidden dynamic structures and dynamic interaction information between nodes, reflecting the actual operation of the network. Therefore, we define the nodes whose shortest distance to node i is less than R as the effective-influence node set $\varphi_{i}$ of node i; the formula for this is as follows:

(4) $$\varphi_{i}\left\{\begin{array}{c} 1\;\;\;\mathrm{if}\sum_{j}^{N}d_{j|i}<R\\ 0\;\;\;\;\;\;\;\;\;\;\;\;\;\;\;\;\;\;\;\;\mathrm{else} \end{array}\right.$$

Here, N denotes the total number of nodes in the network, $d_{j|i}$ represents the shortest distance between nodes i and j, and R signifies the network’s truncation radius. If the specified condition is met, node j is added to the set of nodes with an effective influence $\varphi_{i}$.

### 2.2. Traditional Methods

#### 2.2.1. Degree Centrality (DC)

DC evaluates the significance of a node based on the comparison of its degree. The degree centrality of a node i can be expressed using the following formula:

(5) $${DC}_{i}=\sum_{j}^{N}a_{ij}=k_{i}$$

Here, $k_{i}$ denotes the degree of node i (the number of edges connected to it), and N represents the total number of nodes in the network. Degree centrality measures the number of direct connections a node has, from which the node’s influence on information dissemination or resource flow can be inferred.

#### 2.2.2. Betweenness Centrality (BC)

BC [21] considers a node’s ability to act as a bridge or intermediary in its network and is measured by the number of shortest paths passing through the node, as follows:

(6) $${BC}_{i}=\sum_{j,k\neq i}\frac{N_{jk}(i)}{N_{jk}}$$

where $N_{jk}$ represents the number of shortest paths from node j to node k, and $N_{jk}$(i) is the number of those paths passing through node i. A high betweenness centrality for node i indicates that it plays a more critical role in the network’s information transmission.

#### 2.2.3. Closeness Centrality (CC)

CC [22] measures the average shortest path length from a node to all other nodes. A node with high closeness centrality can access other nodes in the network more quickly, which also means it plays an important role in the network’s structure and information flow. The formula for this is as follows:

(7) $${CC}_{i}=\frac{N-1}{\sum_{j}^{N}d_{ij}}$$

where N represents the number of nodes in the network and $d_{ij}$ is the shortest path distance from node i to node j.

### 2.3. Methods Based on the Gravity Model

#### 2.3.1. Gravity Model (GM)

The GM is defined by drawing an analogy with Newton’s law of universal gravitation. It takes the node’s degree value as the node’s ‘mass’ and the shortest path between nodes as the ‘distance’ between them. The formula for calculating it is as follows:

(8) $${GM}_{i}=\sum_{d_{ij}\leq R,j\neq i}\frac{k_{i}\times k_{j}}{d_{ij}^{2}}$$

where $d_{ij}$ represents the shortest distance between nodes and R is the truncation radius. $k_{i}$ and $k_{j}$ represent the degree values of nodes i and j, respectively, and $d_{ij}$ is the shortest path distance from node i to node j.

#### 2.3.2. Effective Distance Gravity Model (EDGM)

The Effective Distance Gravity Model proposed by Shang Qiuyan et al. [16] considers the effective distance as the distance between nodes. It regards the degree of nodes as their mass, and its formula is as follows:

(9) $${EDGM}_{i}=\sum_{j=1,j\neq i}^{N}\frac{k_{i}\times k_{j}}{D_{j|i}^{2}}$$

where N represents the total number of nodes in the network; $k_{i},k_{j}$ represent the degrees of nodes i and j, respectively; and $D_{j|i}$ represents the effective distance from node i to node j.

#### 2.3.3. Generalized Gravity Model (GGM)

The GGM considers using the degree of a node as its mass to be too simplistic. Instead, it takes the node’s propagation capability as the node’s mass, with the shortest distance as the distance between nodes. Its formula is as follows:

(10) $${GGM}_{i}=\sum_{d_{ij}\leq R,j\neq i}\frac{{Sp}_{i}\times {Sp}_{j}}{d_{ij}^{2}}$$

(11) $${Sp}_{i}=e^{-\alpha C_{i}}\;*\;k_{i}$$

where $d_{ij}$ represents the shortest distance between nodes, R is the truncation radius, and ${Sp}_{i}$ represents the propagation capability of node i. $C_{i}$ is the local clustering coefficient of node i and $k_{i}$ is the degree of node i. When the parameter α is set to 0, the GGM is equivalent to the G model.

## 3. Identification of Important Nodes Based on Local Effective Distance-Integrated Gravity Model

In existing methods for identifying important nodes in complex networks, the comprehensive consideration of node attributes remains inadequate. Studies indicate that neglecting local or topological information when assessing node importance can affect the accuracy of the evaluation results. This paper proposes a novel approach that incorporates the propagation capacity and effective distance of the nodes as key parameters within the gravity model framework to thoroughly consider the local characteristics, global characteristics, positional characteristics, and clustering characteristics of the nodes. However, for large-scale networks, calculating the effective distance between all node pairs is not only time-consuming but also impractical, as nodes typically exert minimal influence on nodes that are far away. Moreover, due to noise accumulation, the interaction strength between distant nodes is difficult to measure accurately. This study addresses these issues by effectively delineating the influence range of nodes, thereby enhancing the efficiency and accuracy of the method.

### 3.1. Algorithm

Step 1: Calculate the effective-influence node set of the nodes

In this step, we calculate and store the effective-influence node set for all nodes in the network. Nodes that are within a distance of less than R from a node are included in the effective-influence node set of that node.

Step 2: Calculate the effective distance

The method for calculating the effective distance between node i and node j is detailed in Section 2.1.1. Specifically, in this step, we compute and store the effective distances between all nodes in the network and the nodes within their effective-influence node set.

Step 3: Calculate the attraction between nodes

The attractiveness between nodes can be determined using the gravitational formula, leading to the calculation of the propagation capability and effective distance of the nodes. A node’s propagation capability is derived from its degree, K-Shell value, and local clustering coefficient. Inspired by the generalized gravity model, we recognize that when nodes have the same degree, the closeness of a node to its surrounding nodes affects its propagation capability.

Building on this, it is evident that when two nodes have the same degree of closeness with their surrounding nodes, the node located closer to the core of the network is more important, indicating that a node’s position within the network topology also affects its propagation capability. The specific calculation formula for this is as follows:

(12) $${W_{interaction}}_{i,j}=\frac{{sp}_{i}\times {sp}_{j}}{D_{j|i}^{2}}$$

where $D_{j|i}$ is the effective distance from node i to j and ${sp}_{i}$ represents the propagation capability of node i, the specific formula for which is as follows:

(13) $${sp}_{i}=e^{-C_{i}}(\frac{k_{i}}{k_{max}}+\frac{{ks}_{i}}{{ks}_{max}})$$

where $C_{i}$ is the local clustering coefficient of node i, $k_{i}$ is the degree of node i, $k_{max}$ is the maximum degree in the network, ${ks}_{i}$ is the K-Shell value of node i, and ${ks}_{max}$ is the maximum K-Shell value in the network.

Step 4: Calculate the importance of the nodes

When calculating the importance of a node, the gravitational forces between that node and the nodes within its effective-influence node set should be summed. The specific formula for this is as follows:

(14) $${IEDG}_{i}=\sum_{j\epsilon \varphi_{i}}{W_{interaction}}_{i,j}=\sum_{j\epsilon \varphi_{i}}\frac{{sp}_{i}\times {sp}_{j}}{D_{j|i}^{2}}$$

where $\varphi_{i}$ is the effective-influence node set of node i and ${IEDG}_{i}$ is the importance of node i.

### 3.2. Example

**Figure 1:**
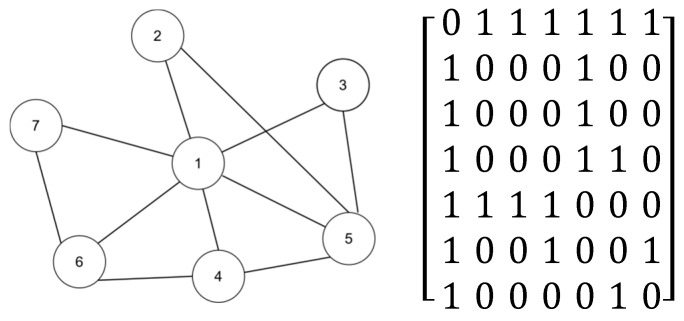
A simple network and its adjacency matrix.

Figure 1 presents an example diagram that includes a simple network and its corresponding adjacency matrix. Initially, we explain the working principle of our algorithm by calculating the LEDGM centrality index for node 2, and we then demonstrate the effectiveness of the effective-influence node set calculation.

The following section outlines the steps required for calculating the LEDGM:

Step 1: Obtain the effective-influence node set of node 2

As shown in Figure 1, the diameter of the network is 2, so its truncation radius is 1. By comparing whether the shortest distance to node 2 is less than the truncation radius, the effective-influence node set of this node can be obtained $\phi_{2}$, $\phi_{2}=\{1,5\}$.

Step 2: Calculate the effective distance between node 2 and its effective-influence node set

Using the formula in Section 2.1.1, we can calculate the effective distance between node 2 and other nodes in its effective-influence node set, using the following specific calculation process:

$$\begin{array}{cc} D_{1|2} & =1-\log_{2}P_{1|2}\\ & =1-\log_{2}\frac{1}{2}\\ & =2 \end{array}\;\begin{array}{cc} D_{5|2} & =1-\log_{2}P_{5|2}\\ & =1-\log_{2}\frac{1}{2}\\ & =2 \end{array}$$

Step 3: Calculate the attraction between node 2 and its effective-influence node set

The specific method for calculating the attraction between node 2 and node 1 is as follows:

$${W_{interaction}}_{2,1}=\frac{v_{2}\times v_{1}}{D_{1|2}^{2}}\;v_{2}=e^{-1}(\frac{2}{6}+\frac{2}{2})=0.4905\;v_{1}=e^{-\frac{1}{3}}(\frac{6}{6}+\frac{2}{2})=1.4331\;{W_{interaction}}_{2,1}=0.1757$$

The attraction between node 2 and node 5 can be obtained using the same method.

$${W_{interaction}}_{2,5}=0.1240$$

Step 4: Calculate the importance of node 2

Using the formula in Step 4 of Section 3.1 for calculation, the specific calculation of the importance of node 2 is as follows:

$${IEDG}_{2}=\sum_{j\epsilon \varphi_{i}}{W_{interaction}}_{2,j}=0.2997$$

In order to prove the validity of the effective influence set, Table 1 shows whether the effective-influence node set is used to calculate the importance index of each node in a complex network. In the table, the LEDGM involves a calculation method that uses the effective-influence node set, while R-LEDGM involves a calculation method that removes the effective-influence node set. A straightforward calculation reveals that without the effective-influence node set, the number of computations required to determine the effective distances between all node pairs in the network is 42, which is equivalent to n × (n − 1). However, with the effective-influence node set, the number of computations is reduced to 20. This reduction significantly lowers the time complexity of the algorithm. Additionally, by comparing the data in Table 1, it is evident that the nodes within the effective-influence node set play a predominant role in the calculation of node importance.

## 4. Experiments and Data

This chapter aims to validate the feasibility and superiority of our proposed method by conducting four different experiments on six real-world networks and comparing its results with those of traditional centrality methods and other similar approaches. Specifically, in Section 4.1, we detail the characteristics of these six real-world network datasets, including the number of nodes and the number of edges in the networks, the average degree of the networks, and the networks’ propagation threshold. In Section 4.2, we employ traditional methods (such as degree centrality (DC), closeness centrality (CC), betweenness centrality (BC), and K-Shell (KS) methods), as well as other methods similar to ours (such as GM, EDGM, GGM, and our proposed LEDGM method), to rank the top 10 nodes in these six networks. In Section 4.3, we utilize the SI (Susceptible–Infected) model and, based on the ranking results of the different methods, select the top ten nodes as the initially infected nodes to verify and analyze the changes in the model’s contagion capabilities under different initial node selections. Additionally, in Section 4.4, we compare the time required for our method and the EDGM method to obtain node influence rankings for the same dataset. In Section 4.5, by comparing the ranking results of the SI model with other methods, we analyze the changes in Kendall’s tau correlation coefficient under different propagation probabilities. Finally, in Section 4.6, the performance of the LEDGM method is evaluated on Erdős–Rényi networks with different node scales but identical topological structures. The results demonstrated that the LEDGM method can effectively adapt to varying network sizes.

### 4.1. Datasets

In this paper, we utilize six datasets for our experiments, including Jazz [23], NS [24], Email [25], EEC [26], PB [27], and USair [28]. These include two communication networks (Email, EEC), a transportation network (USair), a social network (PB), and two collaboration networks (Jazz, NS). The Email network describes the communication patterns occurring among researchers via email; the EEC network represents the electronic communication network among members of European research institutions; the Jazz network illustrates the cooperation among jazz musicians; the NS network is a network of scientists collaborating and working together; the USair network is the transportation network of American air travel; and the PB network is a hyperlink network representing the relationships between American political blogs. Selecting these datasets, which come from different domains, ensures the comprehensiveness and generalizability of our experimental results.

Table 2 presents detailed information about the six networks, including the total number of network nodes N, the number of network edges E, the average shortest distance <d> between nodes, the average degree <k> of the nodes, the network clustering coefficient C, and the network propagation threshold $\beta_{th}$.

### 4.2. Experiment 1: Top Ten Nodes

In this experiment, we conducted a comparative analysis of the similarity among the top ten nodes identified by eight different methods across six networks, aiming to reveal the similarities and differences between these methods. The eight methods include our proposed LEDGM method; the traditional methods DC, BC, CC, and KS; and methods similar to ours, such as GM, EDGM, and GGM. Since each method considers different node characteristics, there are differences in the ranking lists they generate. The number of recurring nodes can, to some extent, reflect the effectiveness of our method. It is important to note that due to significant differences in the characteristics considered by the KS decomposition method compared to the others, we did not compare the similarity of its ranking to that of the LEDGM method.

For detailed ranking results, please refer to Table 3, Table 4, Table 5, Table 6, Table 7 and Table 8. In the Email network, the CC and GGM methods identified the same top ten nodes as the LEDGM method did. Other methods shared 7 to 8 nodes with the LEDGM method, a number lower than that of the CC and GGM methods. In the EEC network, all methods showed a high similarity with the nodes identified by the LEDGM method, with the CC and GGM methods sharing 9 nodes with the LEDGM method. In the Jazz network, the BC and GGM methods had the fewest common nodes with the LEDGM method, while other methods had between 7 and 8 common nodes. In the NS network, the BC and CC methods had the fewest common nodes with the LEDGM method, only 5, while the GGM method had slightly more, and the other methods had between 7 and 8 common nodes. In the USair network, the DC method identified the same nodes as the LEDGM method did, while the BC method had the lowest number of common nodes with the LEDGM method, 6, and other methods had between 8 and 9 nodes. In the PB network, the DC method identified the same nodes as the LEDGM method did, and the other methods all had 9 nodes in common with the LEDGM method. By analyzing the tabular data, we found that the LEDGM method had a high number of nodes that were consistent with the other methods across the different networks, indicating its good adaptability and confirming the rationality of our proposed method. Furthermore, our proposed method performed similarly to other methods across different networks, suggesting that the LEDGM method can effectively integrate global and local characteristics as well as static and dynamic information.

### 4.3. Experiment 2: SI Model

The SI model [29] is a traditional epidemic model used to simulate the spread of infectious diseases in networks to assess the propagation capability of nodes within the network. In the SI model, nodes are divided into two states: (1) susceptible (S); (2) infected (I). The specific propagation process is as follows: infected nodes I spread the disease to susceptible nodes S at a certain infection rate β, after which susceptible nodes S become infected nodes, and infected nodes I remain unchanged. Throughout this process, the total number of nodes N in the complex network remains constant (N = S + I). The faster the increase in the number of infected nodes, the more influential the source of infection is considered to be.

**Figure 2:**
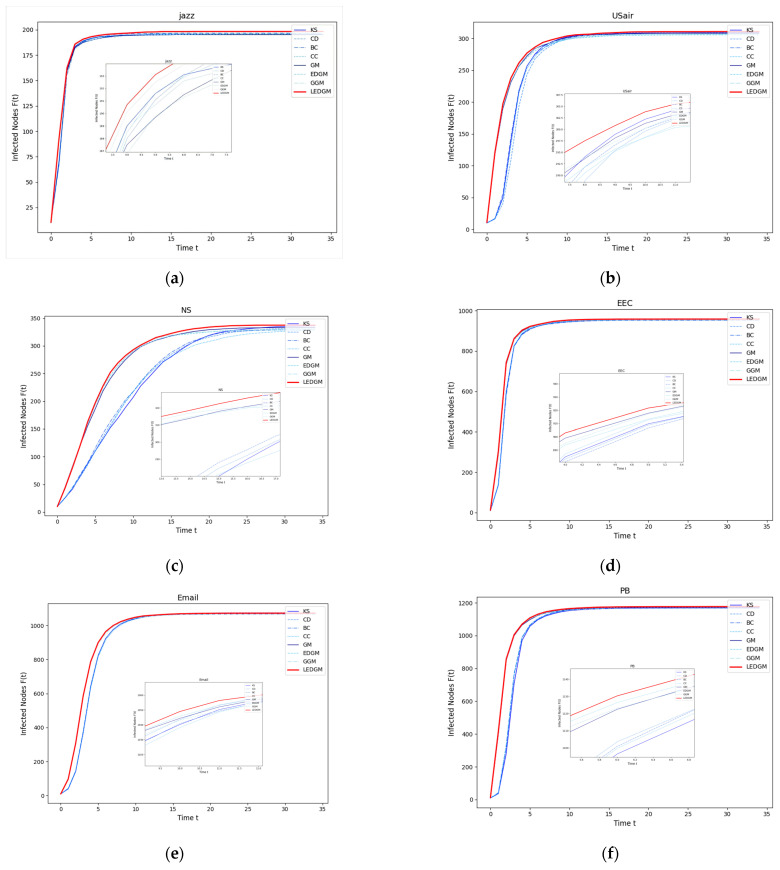
This figure illustrates the infection performance of the top ten important nodes, selected by eight methods, across six different networks: (**a**) The infection performance of the top ten nodes in the Jazz network. (**b**) The infection performance of the top ten nodes in the USair network. (**c**) The infection performance of the top ten nodes in the NS network. (**d**) The infection performance of the top ten nodes in the EEC network. (**e**) The infection performance of the top ten nodes in the Email network. (**f**) The infection performance of the top ten nodes in the PB network.

In this experiment, we selected the top ten nodes identified by the various methods used in Section 4.2 as the initial infected nodes, with the remaining nodes in the network considered to be susceptible nodes. These infected nodes infect surrounding susceptible nodes at an infection rate of β = 0.2. To ensure the objectivity of the experimental results, each experiment was conducted independently 100 times, and the average outcomes are presented in Figure 2. We observed that the higher the importance of a node within the network, the faster the rate of increase in the number of infected individuals, and, consequently, the greater the total number of infected individuals at the end of the experiment.

As shown in Figure 2, in the six networks, the LEDGM method’s infection growth rate and maximum infected nodes are better than those of the other seven methods. Figure 2c,f indicate that gravity-model-based methods outperform similarity-based ones in large networks, with the LEDGM being more effective than the other three gravity-model-based methods. This is because the LEDGM considers the nodes’ local, global, positional, and clustering information for a more comprehensive assessment of their spreading ability and importance.

Experiments on six real-world networks show that although the LEDGM may not be the best in all networks, it has significant advantages in most, especially compared to the GM, GGM, and EDGM. This highlights the LEDGM’s superiority and strong versatility across different types of network.

### 4.4. Experiment 3: Validate the Role of the Effective-Influence Node Set

In this experiment, we analyze the role of the effective-influence node set. By comparing the time taken by two methods, we aim to show its superiority in reducing the algorithms’ time complexity.

From an algorithmic perspective, the effective-influence node set significantly reduces time complexity. Although the effective distance better measures node interactions in complex networks, enhancing analysis efficiency and model predictability, its calculation requires assessing all possible paths between node pairs, resulting in high time complexity O$\left(n^{3}\right)$. This makes methods using the effective distance computationally expensive, especially in large-scale networks.

To tackle this issue, the LEDGM method introduces an effective-influence node set. It uses a screening algorithm to filter out nodes that significantly impact the target node, reducing the number of node pairs undergoing effective distance calculation. This screening algorithm has a time complexity of O$\left(n^{2}\right)$, which greatly reduces the time cost of computing effective distances between network nodes.

In networks where node proximity is not obvious, nodes have more “distant relatives” that are far away and have a negligible influence on the target node. The screening of the effective-influence node set can further reduce the number of distance calculations required, boosting algorithm efficiency. Table 9 shows the specific experimental performance of two different methods.

We performed an experimental analysis of the role of effective-influence node sets in reducing algorithmic time complexity. The hardware used in this experiment was an Intel^®^ Core™ 12th Gen i3-12100F processor with a clock speed of 3.30 GHz. The software environment was Python 3.12.3. Table 9 shows that in all six real-world networks, the method that used the effective-influence node set was more efficient than that without it. It reduced experimental time consumption by 57.91% in the best-performing network and by 13.28% in the worst-performing one. By analyzing the average shortest path length, network diameter, and global clustering coefficient, we found that the Email and USair networks have weak node connectivity and longer paths. This explains why the effective-influence node set is more effective in these networks.

By filtering out nodes with a significant impact on the target node, the effective-influence node set reduced the number of node pairs undergoing effective distance calculation. This lowered the algorithm’s time complexity and made using the effective distance feasible in large-scale networks. Thus, the LEDGM method achieved a significant improvement in algorithmic efficiency while maintaining high accuracy.

### 4.5. Experiment 4: Kendall’s Coefficient

In this experiment, we used Kendall’s coefficient [30] to measure the correlation between the ranking results of different methods and the node ranking results obtained from the SI model, thereby assessing the accuracy of the node importance ranking results of our proposed method and other related methods. We assume that there are two sequences X and Y, each containing N nodes, where X = ($x_{1},x_{2}$, …, $x_{n}$) and Y = ($y_{1},y_{2}$, …, $y_{n}$). Then, a new sequence XY is constructed, where XY = (($x_{1},y_{1}$), ($x_{2},y_{2}$), …, ($x_{n},y_{n}$)), meaning the elements of XY are the results that correspond to one-to-one connections between the elements of X and Y. In the sequence XY, for any pair of elements ($x_{i},y_{i}$) and ($x_{j},y_{j}$), if $x_{i}<x_{j}$ and $y_{i}<y_{j}$, or $x_{i}>x_{j}$ and $y_{i}>y_{j}$, then this pair is considered concordant; if $x_{i}<x_{j}$ and $y_{i}>y_{j}$, or $x_{i}>x_{j}$ and $y_{i}<y_{j}$, then this pair is considered discordant; if $x_{i}=x_{j}\;and\;y_{i}=y_{j}$, then this pair is considered neither concordant nor discordant. The expression for Kendall’s coefficient tau is

(15) $$\tau =\frac{2(n_{a}-n_{b})}{N(N-1)}$$

where the number of concordant pairs and discordant pairs are denoted by $n_{a}$ and $n_{b}$, respectively. The value of τ ranges from −1 to 1, with values closer to 1 indicating a higher positive correlation and values closer to −1 indicating a higher negative correlation.

**Figure 3:**
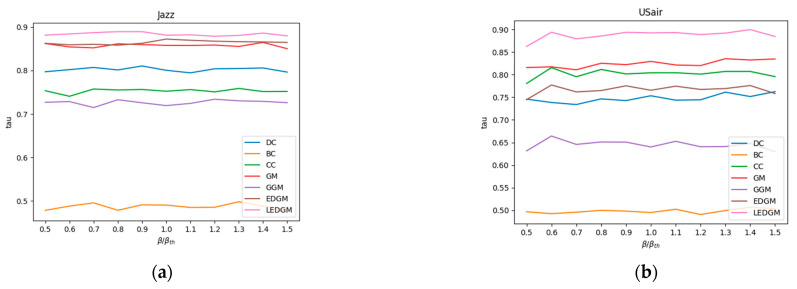

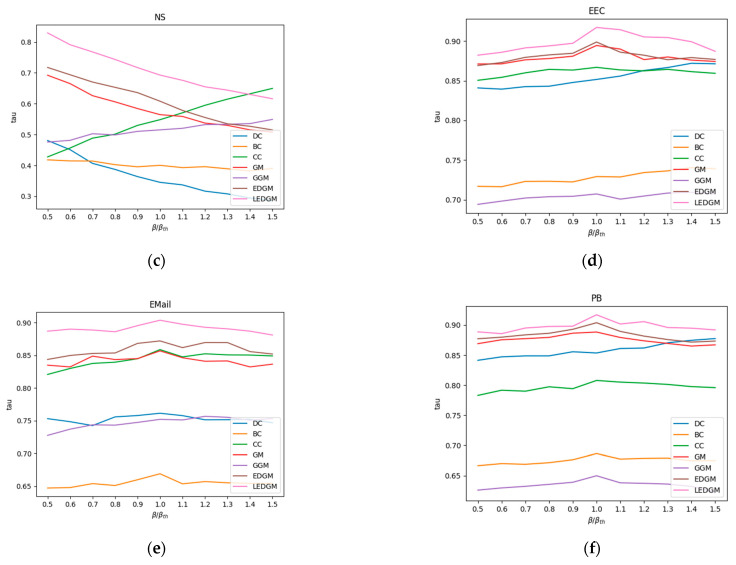
This figure shows the changes in the Kendall coefficient between the rankings generated by seven methods and the standard node rankings produced by the SI model at different infection rates: (**a**) Kendall’s coefficient of various methods, at different infection rates, in the Jazz network. (**b**) Kendall’s coefficient of various methods, at different infection rates, in the USair network. (**c**) Kendall’s coefficient of various methods, at different infection rates, in the NS network. (**d**) Kendall’s coefficient of various methods, at different infection rates, in the EEC network. (**e**) Kendall’s coefficient of various methods, at different infection rates, in the Email network. (**f**) Kendall’s coefficient of various methods, at different infection rates, in the PB network.

In this experiment, we utilized the ranking sequences generated by the SI model in Section 4.3 as a benchmark to assess the accuracy of the ranking sequences produced by the new method we have proposed. When generating the SI model’s ranking sequences, each node in the network was selected as the initial infected node in a separate simulation. To ensure the reliability of the simulation results, each simulation was independently executed 100 times, and the results were averaged to obtain a standard ranking of the nodes’ influence. We employed the Kendall coefficient to measure the correlation between the standard ranking sequences of nodes created by the SI model and those generated by other methods, thereby assessing the accuracy of those methods. The methods compared include DC, BC, CC, GM, GGM, and EDGM. To ensure the objectivity and validity of the experiment, we adjusted the infection probability β in the SI model and conducted simulation experiments, repeating each simulation 100 times and averaging the results to evaluate the effectiveness of different comparison methods under varying infection probabilities. The average results of the experiments are shown in Figure 3. A higher Kendall coefficient indicates a higher correlation with the sequences produced by the SI model, thereby demonstrating the superior performance of the method in terms of accuracy.

By analyzing the data presented in Figure 3, we observed that the LEDGM method consistently ranked first across all six real-world networks. In the Jazz and PB networks, the performance of the EDGM method was close to that of the LEDGM method, yet slightly inferior. We attribute these experimental results to the LEDGM method’s ability to adapt to the network’s topological structure and effectively integrate multidimensional information about the network, thereby accurately capturing the true influence of nodes within the network. Combining the experimental results from the six real-world networks, we conclude that the LEDGM method demonstrates significant superiority over the other methods studied.

### 4.6. Experiment 5: Erdős–Rényi Networks with a Controlled Node Scale

The first four experiments demonstrate the general applicability and effectiveness of the LEDGM method across different network topologies. To further explore the performance of the LEDGM method in networks of varying sizes but with the same topological structure [31], we conducted additional experiments on artificially generated random Erdős–Rényi [32] networks with node scales of 100, 500, 1000, and 2000. The network density was controlled by adjusting the edge generation probability. Specifically, for the network with 100 nodes, the edge generation probability was set to 0.05; for the networks with 500 and 1000 nodes, it was set to 0.01; and for the network with 2000 nodes, it was reduced to 0.005. This approach aimed to prevent overly high network densities, which could lead to rapid global infection and thus compromise our ability to effectively evaluate the performance differences between the methods using the SI model test.

**Figure 4:**
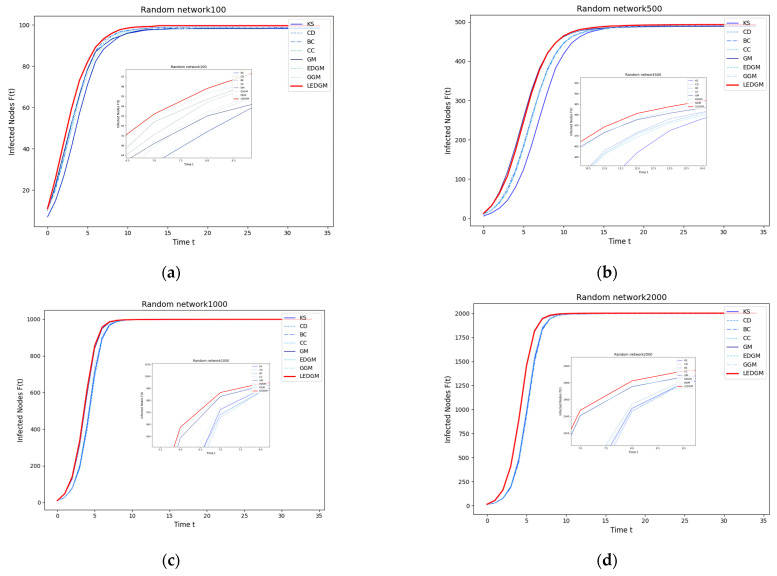
This figure illustrates the infection capability of the top 10 nodes selected by eight methods from four Erdős–Rényi networks of different scales: (**a**) shows the infection capability of the top 10 nodes in an Erdős–Rényi network with 100 nodes; (**b**) in one with 500 nodes; (**c**) in one with 1000 nodes; and (**d**) in one with 2000 nodes.

By comparing Figure 4a–d, it can be seen that the LEDGM method performs well in Erdős-Rényi networks of four different scales. This indicates that the performance of the LEDGM method does not depend on the network size but can adapt to random network topologies of various scales. Additionally, the data in Table 10 further confirm the efficiency and universality of the effective-influence node set across different network sizes. In the network with 2000 nodes, the introduction of the effective-influence node set in the LEDGM method achieved an efficiency improvement of 82.19%. This result highlights the ability of the effective-influence node set to significantly reduce time complexity in large-scale networks, thereby verifying its high efficiency and universality across different network scales. In summary, the LEDGM method not only performs well in networks of different topological structures but also shows strong adaptability and efficiency advantages in networks of the same topological structure but different scales, providing a reliable tool for complex network analysis.

## 5. Conclusions

In order to identify important nodes in complex networks more efficiently and accurately, we propose a method named the LEDGM. This method includes various attribute features of the nodes, characterizing their propagation capabilities by synthesizing node attribute information, thereby effectively identifying influential nodes within the network. Furthermore, the LEDGM method enhances computational efficiency by employing an effective-influence node set, reducing redundant calculations of the effective distances between nodes. Through the analysis of experiments based on the SI disease spread model across six real-world network datasets, we found that the LEDGM method shows great potential in areas such as information transmission, social networking, and road transportation. Compared to seven other methods, the nodes selected by the LEDGM method exhibit stronger propagation capabilities while the model itself showed stronger adaptability across different datasets, thereby proving its effectiveness and superiority. Concurrently, through the analysis of the time efficiency experiments, we found that the LEDGM method has a distinct advantage over the EDGM method in terms of time efficiency.

Although the LEDGM method has performed excellently in identifying important nodes and has also performed well in reducing time complexity, we must also recognize that if we can find the optimal balance between improving the method’s accuracy and reducing its time complexity, the capability and applicability of the LEDGM method will be further enhanced. We recognize that the judicious and skillful use of multi-attribute node information can uncover deeper network node information and hidden topological structures. Therefore, exploring more advanced feature fusion methods will be a focal point of our future research.

## Figures and Tables

**Table 1:** A comparison of whether to use the effective-influence node set to calculate the importance index of each node in a complex network. Here, LEDGM denotes the calculation method that uses the effective-influence node set, while R-LEDGM denotes the calculation method that removes the effective-influence node set.

Node	Node 1	Node 2	Node 3	Node 4	Node 5	Node 6	Node 7
LEDGM	0.4485	0.2997	0.2997	0.3704	0.3577	0.3105	0.2702
R-LEDGM	0.4485	0.3442	0.3442	0.4088	0.3940	0.3594	0.3195

**Table 2:** Topological features of six real networks. N represents the total number of nodes in the network, and E represents the total number of edges. The average degree is represented by <k>. The average shortest path length is <d>. The clustering coefficient is C. The threshold propagation rate is $\beta_{th}$.

Network	N	E	<k>	<d>	C	$\beta_{th}$
Jazz	198	2742	27.6970	2.3235	0.6334	0.0266
USair	332	2126	12.8072	2.7381	0.6252	0.0231
NS	379	914	4.8232	6.0419	0.7981	0.1424
EEC	986	16,064	32.5841	2.5869	0.4070	0.0135
Email	1133	5451	9.6222	3.6060	0.2541	0.0565
PB	1222	16,714	27.3552	2.7375	0.3600	0.0125

**Table 3:** The top 10 nodes, obtained through eight different methods, in the Jazz network.

Rank	Jazz							
	DC	BC	CC	KS	GM	GGM	EDGM	LEDGM
1	7	7	7	0	7	7	7	99
2	99	154	99	3	99	99	99	7
3	3	99	130	4	3	130	3	130
4	130	185	193	8	130	3	130	3
5	193	130	68	31	193	68	193	128
6	128	135	3	32	79	193	79	79
7	79	126	31	41	128	161	68	31
8	161	59	52	64	68	185	128	4
9	68	27	110	79	161	110	161	68
10	76	68	161	80	52	52	52	193

**Table 4:** The top 10 nodes, obtained through eight different methods, in the USair network.

Rank	USair							
	DC	BC	CC	KS	GM	GGM	EDGM	LEDGM
1	117	117	117	200	117	117	117	117
2	260	7	260	149	260	260	260	260
3	254	260	66	292	254	254	254	254
4	181	200	254	300	181	181	181	181
5	151	46	200	231	151	151	229	151
6	229	181	181	257	229	165	165	165
7	165	254	46	178	165	229	66	229
8	66	151	247	66	66	66	111	66
9	111	312	165	111	111	200	146	200
10	200	12	111	117	146	46	200	111

**Table 5:** The top 10 nodes, obtained through eight different methods, in the NS network.

Rank	NS							
	DC	BC	CC	KS	GM	GGM	EDGM	LEDGM
1	3	25	25	3	3	25	3	4
2	25	50	94	4	4	3	4	3
3	4	168	50	15	25	4	25	15
4	15	94	230	14	15	50	15	25
5	66	66	99	44	14	94	14	0
6	69	4	51	45	94	230	50	230
7	94	230	4	46	50	51	230	50
8	14	99	43	175	66	7	66	69
9	112	43	233	176	230	168	69	94
10	50	65	296	69	69	66	51	44

**Table 6:** The top 10 nodes, obtained through eight different methods, in the EEC network.

Rank	EEC							
	DC	BC	CC	KS	GM	GGM	EDGM	LEDGM
1	162	160	160	283	160	160	160	160
2	121	86	82	21	121	86	121	82
3	82	5	121	106	82	82	82	121
4	107	82	107	128	107	121	107	86
5	86	121	62	114	62	62	62	62
6	62	107	86	249	86	107	86	107
7	434	13	434	210	434	13	434	13
8	13	377	166	303	166	5	166	64
9	166	62	249	371	249	64	249	434
10	183	64	64	212	183	166	183	166

**Table 7:** The top 10 nodes, obtained through eight different methods, in the Email network.

Rank	Email							
	DC	BC	CC	KS	GM	GGM	EDGM	LEDGM
1	104	332	332	298	104	22	104	104
2	332	104	22	388	322	104	332	332
3	41	22	104	433	41	332	41	22
4	22	577	41	551	22	232	40	41
5	15	75	40	570	15	40	232	232
6	40	232	75	725	40	134	75	40
7	195	134	232	755	195	41	15	134
8	232	40	51	787	232	51	195	75
9	75	354	134	884	75	75	51	51
10	20	41	377	885	20	377	134	377

**Table 8:** The top 10 nodes, obtained through eight different methods, in the PB network.

Rank	PB							
	DC	BC	CC	KS	GM	GGM	EDGM	LEDGM
1	126	671	837	126	126	126	126	126
2	837	126	126	581	837	837	837	837
3	671	767	496	99	47	671	47	496
4	47	837	47	565	496	767	496	47
5	496	496	889	345	671	496	671	767
6	767	1177	565	499	565	47	565	565
7	1005	47	767	300	1005	1177	1005	671
8	565	781	921	411	921	1005	767	1005
9	921	921	1177	382	767	921	921	1177
10	1177	565	671	36	889	889	889	921

**Table 9:** Comparison of time efficiency between LEDGM method and EDGM method.

Datasets	LEDGM (s)	R-LEDGM (s)	Enhancement Effect
PB	48,349.0783	55,751.6026	13.28%
EEC	20,938.0385	36,986.7475	43.39%
Email	8246.0774	19,593.4822	57.91%
NS	333.3476	492.8544	32.36%
USair	142.8616	328.2217	56.47%
Jazz	96.8969	134.0255	27.70%

**Table 10:** Comparison of the time efficiency of the LEDGM and R-EDGM methods on Erdős–Rényi networks with different node scales.

Node Scale	LEDGM (s)	R-LEDGM (s)	Enhancement Effect
100	6.1920	12.6436	51.03%
500	25.8653	86.9887	70.26%
1000	39.4167	92.6506	57.45%
2000	68.1203	382.3989	82.19%

## Data Availability

Data is contained within the article.

## Abbreviations

The meanings of the acronyms and symbols in this paper are as follows:

**Symbol**	**Implication**
$D_{j|i}$	Effective distance from node i to node j
$d_{ij}$	Shortest distance from node i to node j
C	Local clustering coefficient
R	Truncation radius
φ	Effectively affects the node set
N	Number of network nodes
k	Degree of a node
ks	Node K-Shell value
$k_{max}$	Maximum value of the network
${ks}_{max}$	Maximum K-Shell value of the network
sp	Node propagation capability value
DC	Degree centrality
BC	Betweenness centrality
CC	Closeness centrality
KS	K-Shell method
GM	Gravity model
EDGM	Effective distance gravity model
GGM	Generalized gravity model
